# Functional Integration of Adult-Born Hippocampal Neurons after Traumatic Brain Injury

**DOI:** 10.1523/ENEURO.0056-15.2015

**Published:** 2015-09-28

**Authors:** Laura E. Villasana, Kristine N. Kim, Gary L. Westbrook, Eric Schnell

**Affiliations:** 1 Department of Anesthesiology and Perioperative Medicine, Oregon Health & Science University, Portland, Oregon 97239; 2 VA Portland Health Care System, Portland, Oregon 97239; 3 The Vollum Institute, Oregon Health & Science University, Portland, Oregon 97239

**Keywords:** adult neurogenesis, functional integration, hippocampus, maturation, synaptic integration, traumatic brain injury

## Abstract

Traumatic brain injury (TBI) increases hippocampal neurogenesis, which may contribute to cognitive recovery after injury. However, it is unknown whether TBI-induced adult-born neurons mature normally and functionally integrate into the hippocampal network. We assessed the generation, morphology, and synaptic integration of new hippocampal neurons after a controlled cortical impact (CCI) injury model of TBI. To label TBI-induced newborn neurons, we used 2-month-old POMC-EGFP mice, which transiently and specifically express EGFP in immature hippocampal neurons, and doublecortin-CreER^T2^ transgenic mice crossed with Rosa26-CAG-tdTomato reporter mice, to permanently pulse-label a cohort of adult-born hippocampal neurons. TBI increased the generation, outward migration, and dendritic complexity of neurons born during post-traumatic neurogenesis. Cells born after TBI had profound alterations in their dendritic structure, with increased dendritic branching proximal to the soma and widely splayed dendritic branches. These changes were apparent during early dendritic outgrowth and persisted as these cells matured. Whole-cell recordings from neurons generated during post-traumatic neurogenesis demonstrate that they are excitable and functionally integrate into the hippocampal circuit. However, despite their dramatic morphologic abnormalities, we found no differences in the rate of their electrophysiological maturation, or their overall degree of synaptic integration when compared to age-matched adult-born cells from sham mice. Our results suggest that cells born after TBI participate in information processing, and receive an apparently normal balance of excitatory and inhibitory inputs. However, TBI-induced changes in their anatomic localization and dendritic projection patterns could result in maladaptive network properties.

## Significance Statement

Post-traumatic neurogenesis may contribute to recovery after traumatic brain injury (TBI), but the functional integrity of neurons generated after injury is critical and has not yet been examined. We used multiple lines of transgenic mice to label cells born after TBI in order to study their maturation and synaptic integration. Although cells born after TBI had significantly altered morphology and anatomic localization, they functionally integrated into the hippocampal circuit. Our data indicate that these adult-born neurons contribute to network connectivity after TBI, although changes in their morphology and dendritic projection patterns may alter their functional capacity.

## Introduction

In mammals, the generation of adult-born hippocampal neurons persists into late adulthood (van Praag et al., 2005; Zhao et al., 2008; Spalding et al., 2013) and is influenced by multiple environmental contingencies, such as learning (Gould et al., 1999), exposure to enriched environments (Kempermann et al., 1997), and exercise (van Praag et al., 1999). Hippocampal neurogenesis also increases in response to various neurological insults, including hypoxia (Zhu et al., 2005; Varela-Nallar et al., 2014), ischemia (Liu et al., 1998; Jin et al., 2001), seizures (Parent et al., 1997; Scharfman et al., 2000), and traumatic brain injury (TBI; Dash et al., 2001; Chirumamilla et al., 2002; Villasana et al., 2014). Because adult-born hippocampal neurons are important for learning and memory (Shors et al., 2001; Dupret et al., 2008; Sahay et al., 2011), post-traumatic hippocampal neurogenesis may compensate for functional deficits resulting from injury and contribute to cognitive recovery. To assay neurogenesis after TBI, prior studies have focused on histochemical approaches in fixed tissue (Dash et al., 2001; Chirumamilla et al., 2002; Rice et al., 2003; Sun et al., 2007; Yu et al., 2008; Blaiss et al., 2011), which precludes functional analysis. Although the axons of granule cells born after TBI project to hippocampal region CA3 (Sun et al., 2007), it remains unknown whether these outputs are functional and, more importantly, whether these cells receive afferent input. Because mature granule cells in the dentate gyrus can be hyperexcitable after TBI (Santhakumar et al., 2000; Gupta et al., 2012), it is possible that cells born after TBI develop similar changes during their maturation.

Thus, to determine whether TBI alters the development and functional integration of adult-born hippocampal neurons, we used two lines of transgenic mice to assess early and late stages of the development and integration of adult-born neurons after a controlled cortical impact (CCI) model of TBI (Dash et al., 2001; Kernie et al., 2001). Proopiomelanocortin-enhanced green fluorescent protein (POMC-EGFP) transgenic mice transiently express GFP in adult-born neurons ∼7-14 d postmitosis, and were used to assess hippocampal neurogenesis at an early stage (Overstreet-Wadiche et al., 2006; Hunt et al., 2012; Villasana et al., 2014). We also used a novel line of transgenic mice, the doublecortin (Dcx)-CreER^T2^ line (Cheng et al., 2011), which, when crossed with a Rosa26-CAG-tdTomato (tdTom) reporter mouse (Madisen et al., 2010), allowed us to permanently and noninvasively pulse-label hippocampal granule cells born after TBI. Our results show that neurons born during post-traumatic neurogenesis survive and functionally integrate into hippocampal circuits, and show apparently normal synaptic properties despite markedly altered location and dendritic morphology.

## Material and Methods

### Animals

All procedures were performed according to the standards of the National Institutes of Health *Guide for the Care and Use of Laboratory Animals* and in compliance with approved institutional animal care protocols. Subjects were 2-month-old male and female heterozygotic POMC-EGFP mice (Overstreet et al., 2004) and Doublecortin-CreER^T2^ mice (generously provided by Dr. Zhi-Qi Xiong, Institute of Neuroscience, Shanghai, China; Cheng et al., 2011) crossed with Rosa26-CAG-tdTomato marker mice (Madisen et al., 2010) and used as double heterozygotic transgenic mice. These complementary mouse lines allowed us to label neurons born during post-traumatic neurogenesis, and to examine them at both early and late time points. As these markers are expressed *in vivo*, this approach was used to analyze the physiological function of adult-born cells in acutely prepared brain slices as well as the morphology of adult-born cells in fixed tissue. Littermates were randomly allocated to either sham injury or CCI treatment. Prior to any quantification, the hippocampus was inspected, and any mice with damage to the dentate gyrus were excluded entirely (*n* = 3 total), and any slices with any damage to the Cornu Ammonis regions (CA1/CA3) of the hippocampus were discarded.

### Controlled cortical impact injury

We used a CCI protocol to induce TBI, as previously described (Kernie et al., 2001) with some modifications. Mice were anesthetized using spontaneously inhaled isoflurane (2%) and mounted on a stereotaxic apparatus. A 4 mm craniotomy incision was made (dura intact) between lambda and bregma, bordered on the right of the midline. A 0.9 mm deformation (4.4 m/s; 800 ms dwell) was made in the exposed cortical area using a 3-mm-diameter sterile stainless steel tip attached to an electromagnetic impactor (ImpactOne, Leica Microsystems). The scalp was then sutured, and mice recovered in a warm padded chamber. Sham mice received the same treatment (anesthetic, scalp incision/closure), with the exception of the craniotomy and impact. Each mouse was individually coded, and the experimenters were blinded for subsequent analyses. All mice survived CCI. Mice were killed 2 or 4 weeks after sham or CCI treatment for POMC-EGFP and DcxCre/tdTom mice, respectively.

### Bromodeoxyuridine injections

Because dendritic development occurs rapidly in immature neurons and can be accelerated by neuronal injury (Overstreet-Wadiche et al., 2006; Niv et al., 2012), bromodeoxyuridine (BrdU) was used to date neurons in POMC-EGFP mice and to allow for comparison of age-matched cells between treatment groups. BrdU (Sigma-Aldrich) was dissolved in warm sterile saline solution (10 mg/ml) and injected at 300 mg/kg, i.p., twice a day for 2 d at 2, 5, or 7 d post-CCI (three separate cohorts). These mice were killed exactly 2 weeks after injury, such that the BrdU-labeled neurons were 12, 9, or 7 d postmitosis, respectively. The dendritic and somatic morphology of BrdU^+^ cells was determined based on their GFP expression.

### Tamoxifen

To pulse label newborn neurons in DcxCre/tdTom mice, mice received 2 daily intraperitoneal injections (7 h apart) of tamoxifen (40 mg/kg in corn oil) for 3 d, starting 6 d after CCI. Previous studies have confirmed that this regimen pulse labels hippocampal granule cells born 2-3 d prior to tamoxifen administration in DcxCreER^T2^/marker mice (Cheng et al., 2011), which we confirmed using BrdU colabeling (300 mg/kg, i.p., two doses 4 h apart on a single day) and Dcx costaining. These mice provide a complementary approach to label adult-born neurons, for although immature neurons are efficiently labeled in POMC-EGFP mice, GFP expression is lost as they mature. In contrast, in DcxCre/tdTom mice, tdTom expression is permanent and can be used to analyze adult-born cells after they have matured. However, tdTom expression takes several days to become bright enough for detailed morphologic assessment of dendritic branching (see Fig. 4 below), and thus the POMC-EGFP animal was used for analysis at early time points.

### Immunohistochemistry

Mice were terminally anesthetized according to institutional animal care and use committee-approved protocols, transcardially perfused with 4% paraformaldehyde in PBS, and post-fixed overnight. Free-floating coronal sections (150 μm thick) were prepared from each mouse and permeabilized in 0.4% Triton in PBS (PBST) for 45 min. Four sections containing the hippocampus (two dorsal: approximately −1.46 and −2.18 mm from bregma; and two ventral: approximately −2.54 and −2.80 mm from bregma) from each mouse were then blocked for 30 min with 10% horse serum in PBST and incubated overnight (4°C) with primary antibody in 1.5% horse serum/PBST. The primary antibodies were rabbit anti-GFP (Alexa Fluor 488 conjugated; 1:400; Invitrogen), rat anti-BrdU (1:500; Abcam), and guinea pig anti-Dcx (1:500; Millipore). Sections incubated with anti-BrdU were first incubated in 2N hydrochloric acid in potassium-PBST for 30 min (37°C), washed twice, and blocked with horse serum, as described above. The BrdU-stained samples were washed in PBST (2× 10 min) the following day and incubated with goat anti-rat (1:400; Rhodamine Red 568, Jackson Laboratories), donkey anti-rat (1:400; Alexa Fluor 488, Invitrogen), or goat anti-guinea pig (1:400; Alexa Fluor 488, Invitrogen) for 2 h at room temperature. The sections were then washed in PBST (2× 10 min) and mounted with DAPI Fluoromount-G (SouthernBiotech) on coded slides.

### Confocal microscopy

Four separate stained slices from the hippocampus of each mouse were imaged with a Zeiss LSM780 confocal microscope using a 10×/0.45 numerical aperture (NA) or 20×/0.8 NA lens. Images were kept coded and subsequently quantified using ImageJ software by an investigator blinded to experimental condition. For POMC-EGFP animals, all GFP^+^ cells in a 10 μm *z*-stack within the middle region of the suprapyramidal blade of the dentate gyrus granule cell layer (GCL), including the subgranular zone (SGZ), were counted and normalized to the imaged GCL volume for each slice ipsilateral and contralateral to injury or sham. In DcxCre/tdTom mice, tdTomato^+^ cells were quantified through the GCL of the entire span of the dentate gyrus/section in a 40-μm-thick *z*-stack and normalized to GCL volume. A greater sample volume was used to quantify adult-born cell density in DcxCre/tdTom mice than in POMC-EGFP mice because of the lower density of labeled cells in these animals. GCL volume was obtained by multiplying the area of the GCL within the quantified span by its depth (distance along the *z*-axis). The dorsal/ventral regions of hippocampus collected for the DcxCre/tdTom mice were similar to those for POMC-EGFP mice, and were kept constant between sham and CCI animals.

To assess cell migration, the distance from the center of each cell body to the SGZ/hilus border was measured for all cells in the middle section of the suprapyramidal blade of the dentate gyrus, as this was representative of the migration of cells throughout the dentate. Ectopic hilar or molecular layer migration was defined as the migrated cell lying outside of the GCL, >10 μm away from the inner border of the SGZ or the outer border of the GCL, respectively. Dendritic tree morphology was obtained by identifying cell bodies in the middle (*z*-direction) of a tissue section, imaging them in their entire *z*-axis using a confocal stack, and tracing them off-line using ImageJ. Cells with dendrites truncated by sectioning were not included. Group data were obtained from five to eight cells from each animal using the ImageJ Sholl analysis plug-in [Ghosh laboratory (http://labs.biology.ucsd.edu/ghosh/software/)]. ImageJ was also used to measure the distance between the soma and the first branch point, the angle between the two farthest dendrites with the vertex at the first apical dendritic branch, and the area contained within the space bordered by the two farthest dendrites seen on the projected image and the edge of the outer molecular layer (OML). Total dendritic distance was calculated as the sum of the length of all dendrites for each cell. tdTom^+^ cells were also categorized based on their morphology into the following four major classes: (1) typical (one apical dendrite directed toward the molecular layer with its first-order branch >10 μm from its cell body); (2) cells with more than one apical dendrite; (3) cells with dendrites that branched proximal to their cell body (<10 μm); and (4) cells with laterally projecting apical dendrites (<30° from the long axis of the GCL).

Spine density was assessed in the inner molecular layer (IML) and OML of the dentate for adult-born cells for POMC-EGFP and DcxCre/tdTom mice, respectively, using images of dendritic segments obtained using a 40×/1.4 NA lens from three to five different cells for each animal. For any given tdTom^+^ cell, spine densities in the inner and middle layers appeared similar to the density in the OML, and were not quantified. For all analyses of sham versus CCI-treated mice as well as ipsilateral and contralateral samples, matching regions of the suprapyramidal blade (middle portion) of the dentate gyrus were used. Sections were also matched on their anteroposterior axis between animals.

### Electrophysiology

Acute hippocampal slices were prepared from mice 2 weeks (POMC-EGFP mice) or 1 month (DcxCre/tdTom mice) after sham or CCI treatment. After deep isoflurane anesthesia, mice received a terminal dose of avertin followed by transcardiac perfusion using an ice-cold choline chloride-based solution. The 300 μm transverse hippocampal slices were cut on a Leica 1200S Vibratome from the hemisphere ipsilateral to the injury/sham treatment, and were allowed to recover in ACSF containing 1.3 mm sodium ascorbate at 34°C for 30 min before transfer to room temperature. The external solution (ACSF) contained the following (in mm): 125 NaCl, 25 NaHCO_3_, 2.5 KCl, 1.25 NaH_2_PO_4_, 2.0 CaCl_2_, 1.0 MgCl_2_, and 25 d-glucose, bubbled with 95% O_2_-5% CO_2_. Individual fluorescent (adult-born) granule cells were identified using combined fluorescence/differential interference contrast imaging. Cells were patched using 3-4 MΩ leaded glass pipettes (WPI), which allowed us to form seals with resistances of >50 GΩ. For single-cell voltage-clamp recordings, the cesium-gluconate-based internal solution contained the following (in mm): 100 gluconic acid, 10 EGTA, 10 HEPES, 17.5 CsCl, 8 NaCl, 2 Mg-ATP, and 0.3 Na-GTP, pH 7.3 (using 50% CsOH), 290 mOsm. Signals were obtained using an Axopatch 200B amplifier, filtered at 5 kHz, and sampled at 10 kHz using IGOR Pro software (WaveMetrics) and a NIDAQ A/D board. Passive cell membrane properties and series resistances were monitored on-line using a −10 mV test pulse. Series resistances ranged from 6 to 20 MΩ and were not significantly different between groups in any experiments.

Synaptic responses were evoked using a bipolar stimulating electrode (FHC Inc.) placed at the middle molecular layer (MML)/OML border (for DcxCre/tdTom cells) or at the IML/MML border (POMC-EGFP cells) ∼100 μm away from the cell. IPSCs were measured while clamping the granule cell at the verified reversal potential for glutamatergic currents (0 mV) in the absence of receptor antagonists. AMPAR- and NMDAR-mediated currents were obtained by recording from cells at −70 and +40 mV in the presence of the GABA_A_ receptor (GABA_A_R) antagonist SR95531 (10 μm), and the relative NMDAR-mediated current was measured as the amplitude of the combined EPSC measured at +40 mV at a latency of 60 ms after stimulation, at which point the AMPAR-mediated current had completely decayed. sEPSCs and mEPSCs were recorded at −70 mV in SR95531 for a minimum of 5 min of continuous recording (per cell) in the absence or presence of TTX, respectively. Events were automatically detected using a rise time-based algorithm with custom-written software in IGOR Pro software, with a threshold amplitude set at 4 pA, followed by manual checking of each event.

Whole-cell current-clamp recordings were performed using a potassium gluconate-based internal solution containing the following (in mm): KGluconate 130, KCl 20, HEPES 10, EGTA 0.1, Mg-ATP 4, and Na-GTP 0.3, pH 7.2. Cells were patched in voltage-clamp mode, and resting membrane potential was determined as the membrane voltage measured in tracking (current = 0) mode immediately after break-in. The junction potential was −8 mV and was uncorrected. Passive cell membrane properties were determined using a −10 mV voltage step in voltage-clamp mode prior to experiments. In current clamp, small amounts of negative current were injected (1-2 pA) if needed to bring all cells to equivalent *V*_m_ = −80 mV for current step experiments. Voltage changes were recorded in response to 2 s current steps of −10 to +50 pA for POMC-EGFP cells, and from −10 to +200 pA for tdTom^+^ cells.& Spike amplitude, width, rise slope, and threshold were measured from single traces using IGOR Pro software.

### Statistics

For all statistical analyses, data were first assessed for normality and homogeneity of variance to determine the use of parametric or nonparametric tests, as indicated in the Results section. Data are expressed as the mean ± SEM. Statistical analyses were conducted using SPSS Statistics (IBM), and group comparisons were considered significant at *p* < 0.05. All figures were generated using Prism Software (GraphPad Software) or IGOR Pro (WaveMetrics). Table 1 lists the tests used and the retrospective power calculation for each reported statistical measurement.

**Table 1:** Statistical table

	Data structure	Type of test	Power
1	Normal distribution	Two-way ANOVA	0.94
2	Normal distribution	Two-way ANOVA	0.24
3	Normal distribution	Two-tailed *t* test	0.87
4	Normal distribution	Two-way ANOVA	0.03
5	Non-normal distribution	Kruskal–Wallis	0.97
6	Non-normal distribution	Dunn’s *post hoc*	0.60
7	Non-normal distribution	Dunn’s *post hoc*	0.50
8	Normal distribution	Fisher’s LSD	0.91
9	Normal distribution	Two-way ANOVA	0.96
10	Normal distribution	Two-way ANOVA	0.44
11	Non-normal distribution	Mann–Whitney *U* test	0.84
12	Non-normal distribution	Mann–Whitney *U* test	0. 12
13	Normal distribution	Three-way repeated-measures ANOVA	0.32
14	Normal distribution	Fisher’s LSD	0.27, 0.78, 0.94, 0.97, 0.34, 0.03, 0.08, 0.11, 0.08, and 0.04 for each branch point
15	Normal distribution	Three-way repeated-measures ANOVA	0.56
16	Normal distribution	Two-tailed *t* test	0.90
17	Normal distribution	Two-tailed *t* test	0.94
18	Normal distribution	Two-tailed *t* test	0.91
19	Normal distribution	Two-tailed *t* test	0.08
20	Normal distribution	Two-tailed *t* test	0.12
21	Normal distribution	Two-tailed *t* test	0.31
22	Normal distribution	Two-tailed *t* test	0.04
23	Normal distribution	Two-tailed *t* test	0.12
24	Normal distribution	Two-tailed *t* test	0.16
25	Normal distribution	Two-tailed *t* test	0.22
26	Normal distribution	Two-tailed *t* test	0.69
27	Normal distribution	Two-tailed *t* test	0.16
28	Normal distribution	Two-tailed *t* test	0.19
29	Non-normal distribution	Mann–Whitney *U* test	0.74
30	Non-normal distribution	Mann–Whitney *U* test	0.54
31	Non-normal distribution	Mann–Whitney *U* test	0.72
32	Normal distribution	Two-way repeated-measures ANOVA	0.99
33	Normal distribution	Two-way repeated-measures ANOVA	0.47
34	Normal distribution	Fisher’s LSD	0.95, 0.87, 0.85, 0.86, and 0.51 for branch points between 10 and 50 μm from the soma; 0.55, 0.90, 0.94, 0.76, 0.86, 0.86, 0.85, and 0.55 for branch points between 150 and 220 μm from the soma
35	Normal distribution	Two-tailed *t* test	0.97
36	Normal distribution	Two-tailed *t* test	0.92
37	Normal distribution	Two-tailed *t* test	0.54
38	Non-normal distribution	Mann–Whitney *U* test	0.64
39	Normal distribution	Two-tailed *t* test	0.09
40	Normal distribution	Two-tailed *t* test	0.06
41	Normal distribution	Two-tailed *t* test	0.03
42	Normal distribution	Two-tailed *t* test	0.04
43	Normal distribution	Two-tailed *t* test	0.06
44	Normal distribution	Two-tailed *t* test	0.06
45	Normal distribution	Two-tailed *t* test	0.05
46	Normal distribution	Two-tailed *t* test	0.22
47	Normal distribution	Two-tailed *t* test	0.27
48	Normal distribution	Two-tailed *t* test	0.04
49	Normal distribution	Two-tailed *t* test	0.06
50	Normal distribution	Two-tailed *t* test	0.12
51	Normal distribution	Two-tailed *t* test	0.05
52	Normal distribution	Two-tailed *t* test	0.04 and 0.06 for PPF 50, 100, and 250 ms
53	Normal distribution	Two-tailed *t* test	0.08
54	Normal distribution	Two-tailed *t* test	0.07
55	Normal distribution	Two-tailed *t* test	0.05
56	Normal distribution	Two-tailed *t* test	0.10

PPF, Paired-pulse facilitation.

## Results

### CCI increases the generation and outward migration of immature newborn neurons early after injury

**Figure 1.**
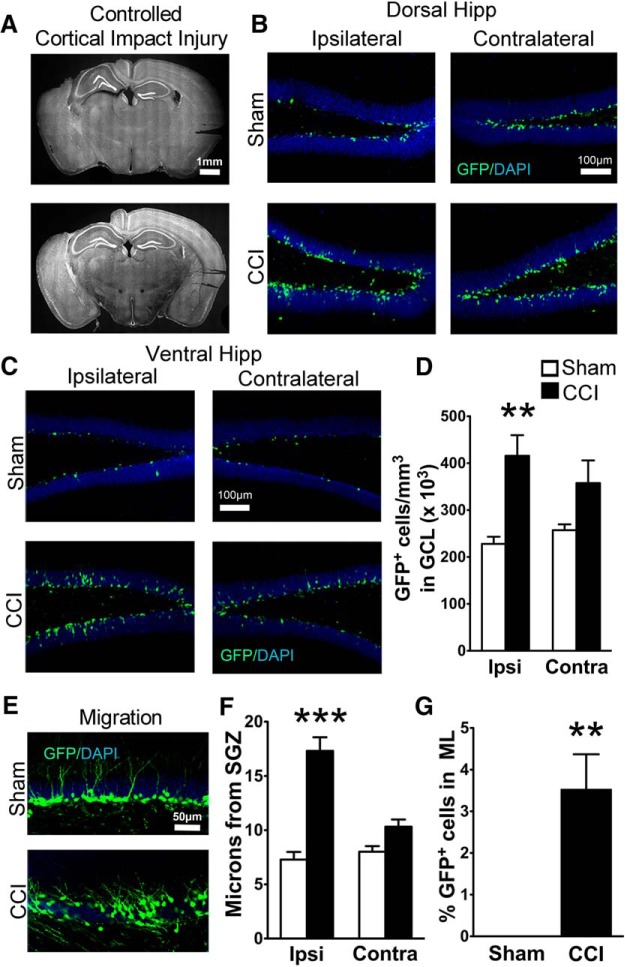
Transgenic POMC-EGFP mice demonstrate CCI-induced neurogenesis and increased dispersion of immature granule cells. ***A***, Representative images of the extent of cortical damage 2 weeks following CCI. The noninjured (contralateral) sides are marked by notches in the cortical tissue placed during processing. ***B***, ***C***, Representative images of GFP^+^ cells in the dorsal (***B***) and ventral (***C***) hippocampus of sham and CCI-treated mice 2 weeks after surgery, both ipsilateral and contralateral to injury. ***D***, CCI-treated mice had more GFP^+^ cells on the injured hemisphere compared to sham mice (**p = 0.0001^3^, *n* = 7 and 8 mice/group). ***E***, Representative images of GFP^+^ cell dispersion in the granule cell layer of the ipsilateral dentate gyrus. ***F***, CCI-treated mice had increased cell migration away from the SGZ on the injured hemisphere compared with sham mice (***p = 0.0001^8^, *n* = 6 mice/group; white and black bars represent sham and CCI-treated mice, respectively). ***G***, A greater percentage of GFP^+^ cells from CCI-treated mice migrated into the molecular layer (ML) of the ipsilateral dentate gyrus (**p = 0.0028^11^, *n* = 6 mice/group).

We used a CCI model of TBI in mice (Fig. 1*A*) to follow the early development of neurons born after injury. We used POMC-EGFP mice because they specifically and transiently express GFP in immature (7- to 14-d-old) hippocampal adult-born neurons, providing both quantitative and qualitative assessments of neurogenesis (Overstreet et al., 2004). Mice were analyzed 2 weeks after injury, so that GFP-positive cells were those generated during the first few days after CCI.

In accordance with prior observations (Dash et al., 2001; Kernie et al., 2001), CCI increased the number of immature adult-born neurons (GFP^+^ cells) in region-matched sections of dentate gyrus (*F*_(1,26)_ = 12.09; *p* = 0.002^1^; *n* = 7, 8 mice per group; Fig. 1*B*,*D*). Although the effect was more pronounced in the ipsilateral hemisphere, there was no significant hemisphere × injury interaction (*p* = 0.26^2^). However, when comparing hemispheres separately between sham and CCI-treated mice using two-tailed *t* tests, a significant difference was observed only on the injured side (*p* = 0.0001^3^). Therefore, further analyses focused on the ipsilateral hemisphere. As CCI occasionally caused morphologic distortion of the dorsal dentate gyrus near the injury (Fig. 1*A*, top), we also analyzed cell density in the ventral hippocampus, which did not have any morphologic alterations. The CCI-induced increase in GFP^+^ cell density was observed throughout the ipsilateral dentate gyrus, as there was no significant interaction between hippocampal region (dorsal vs ventral) and treatment (*p* = 0.83^4^; Fig. 1*B*,*C*). To determine whether the increase in neurogenesis was influenced by sex, we compared the densities of immature adult-born neurons in male and female mice. The overall effect of CCI on the number of adult-born neurons (*p* = 0.0001^5^) was significant in both female (*p* = 0.027^6^) and male mice (*p* = 0.049^7^; female sham injury mice: 238 ± 21 cells/mm^3^, *n* = 3; female CCI injury mice: 497 ± 15 cells/mm^3^, *n* = 3; male sham injury mice: 204 ± 22 cells/mm^3^, *n* = 4; male CCI injury mice: 331 ± 30 cells/mm^3^, *n* = 5).

As adult-born hippocampal granule cells mature and integrate into the hippocampus, they migrate from the subgranular zone toward the molecular layer (Cameron et al., 1993), and this migration can be accelerated by injuries such as seizures (Overstreet-Wadiche et al., 2006). Similar to closed head injury (Villasana et al., 2014), CCI caused a dramatic increase in the outward migration of GFP^+^ neurons born after injury specifically in the injured hemisphere (*p* = 0.0001^8^ in the injured hemisphere; *F*_(1,20)_ = 21.44; *p* = 0.0002^9^, hemisphere × injury interaction, *n* = 6 mice per group; Fig. 1*E*,*F*) . The increased migration was also independent of distance from the injury, as there was no difference between the dorsal and ventral hippocampus in the injured hemisphere (*p* = 0.075^10^, hippocampal region × injury interaction); therefore, these regions were combined for further statistical analysis. However, we still maintained matched dorsal and ventral hippocampal regions between groups, and between ipsilateral and contralateral samples. Post-CCI, a small percentage of newborn neurons had even migrated into the molecular layer, which was rarely observed in sham mice (*p* = 0.0028^11^; Fig. 1*E*,*G*). However, unlike other injury models (Parent et al., 1997; Scharfman et al., 2000), CCI did not increase the percentage of cells that migrated into the hilus (sham injury, 3.6 ± 0.6%; CCI, 5.2 ± 1.5%; *p* = 0.44^12^).

### CCI increases the dendritic complexity of immature neurons born after injury

**Figure 2.**
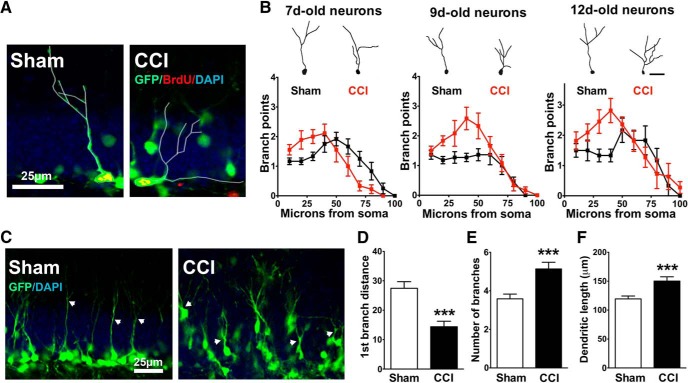
Granule cells born after CCI have accelerated dendritic growth. ***A***, Confocal image of BrdU^+^ cells expressing GFP in POMC-EGFP mice, used to precisely date the birth of cells for morphologic analysis. In this example, BrdU was administered to POMC-EGFP mice 5 d after CCI or sham procedure and perfusion was fixed at 14 d, so that BrdU^+^ cells were 9 d postmitosis. Traced cells are outlined in white. ***B***, Representative images (top) of dendritic tracings of 7-, 9-, and 12-d-old GFP^+^ cells in sham and CCI-treated mice, and the Sholl analyses (bottom) for group data at each time point. In each cohort, CCI increased the number of dendritic branch points of immature cells born during post-traumatic neurogenesis (*p* = 0.0027^13^). Branches also occurred closer to the cell body in CCI-treated mice in each cohort, which was independent of cell age (*p* = 0.76^15^). ***C***, Representative images of GFP^+^ cells and their dendritic outgrowth. White arrows point to the first dendritic branch points. ***D–F***, GFP^+^ cells from CCI treated mice branched closer to their cell body (***p = 0.0012^16^; ***D***), had more cumulative branches (***p = 0.0005^17^; ***E***), and had greater total dendritic length (***p = 0.001^18^; ***F***).

During the first two postmitotic weeks, adult-born granule cells extend apical dendrites through the GCL and into the IML of the dentate gyrus (Zhao et al., 2006). As neuronal insults can alter dendritic maturation of adult-born cells (Overstreet-Wadiche et al., 2006; Jessberger et al., 2007; Niv et al., 2012), we analyzed the arborization of neurons born after CCI. Although GFP^+^ cells constitute a relatively restricted, time-defined cohort of adult-born cells (7-14 d postmitosis), this is a stage of rapid dendritic growth. Thus, we more precisely dated the birth of GFP^+^ cells in POMC-EGFP mice by administering BrdU to mice 2, 5, or 7 d after CCI or sham injury (studied in three separate cohorts of animals). BrdU^+^/GFP^+^ neurons were imaged in three dimensions, and their dendrites were traced at 2 weeks post-CCI for each cohort (Fig. 2*A*). Sholl analysis revealed that CCI altered the dendritic structure of immature neurons (*F*_(9,740)_ = 2.846; *p* = 0.0027^13^; branch points × treatment interaction; Fig. 2*B*), characterized primarily as a selective increase in the number of branches close to the soma (*p* = 0.006, 0.0004, and 0.0001 at 20, 30, and 40 μm from the soma, respectively^14^; *n* = 45 and 31 cells from seven sham- and eight CCI-treated mice with all ages combined; Fig. 2*B*,*C*). There was no interaction between treatment and cell age (*p* = 0.76^15^), and this is reflected by the persistent effect of CCI on dendritic structure in each of the three cohorts of 7-d-old cells (sham, 12 cells from two mice; CCI, 9 cells from two mice), 9-d-old cells (sham, 11 cells from three mice; CCI, 12 cells from three mice), and 12-d-old cells (sham, 6 cells from two mice; CCI, 11 cells from three mice; Fig. 2*B*); therefore, the different cell ages were combined. CCI-treated mice also had shorter distances between their somata and first branch point (*p* = 0.0012^16^; Fig. 2*C*,*D*), more dendritic branches (*p* = 0.0005^17^; Fig. 2*E*), and increased total dendritic length (*p* = 0.001^18^, *n* = 48 cells from six sham mice; 52 cells from six CCI-treated mice; Fig. 2*F*).

### Neurons born during post-traumatic neurogenesis have preserved early electrophysiological maturation

**Figure 3.**
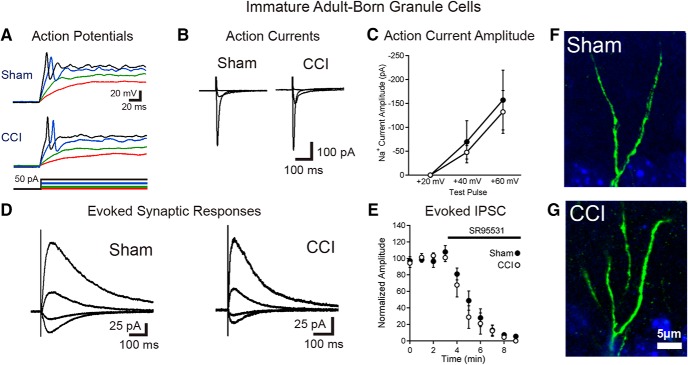
Immature granule cells born after TBI are excitable and integrated into the hippocampal circuitry. ***A***, Current-clamp recordings from GFP-positive immature granule cells born after sham or CCI surgery demonstrate that cells born after TBI fire action potentials during depolarizing current injection. Respective voltage traces during current injections of +5 pA (red), +10 pA (green), +30 pA (blue), and +40 pA (black) for both sham and CCI-treated animals are shown. Summary data are given in the text. ***B***, ***C***, Single-cell, voltage-clamp recordings demonstrate that immature granule cells have similar voltage-dependent action currents. ***B***, Averaged action currents from example cells during steps of +20, +40, and +60 mV from a holding potential of −70 mV. ***C***, Summary data of the amplitude of the action current response in response to the various steps between groups (sham = 7 cells; CCI = 7 cells; *p* = 0.65^54^ with a +40 mV step; *p* = 0.75^55^ with a +60 mV step). ***D***, Synaptic currents elicited in immature adult-born neurons in response to stimulation at the IML/MML border, while voltage clamping the cells to −70, −40, 0, and +40 mV. ***E***, Summary data of response amplitudes of evoked currents (recorded while clamping cells at +40 mV), demonstrating almost complete block of the evoked synaptic response by the GABA_A_R antagonist SR95531 (10 μm; *n* = 5 cells per group). ***F***, ***G***, High-power imaging of dendrites from newborn neurons in sham and CCI-treated mice show no evidence of dendritic spines on immature GFP^+^ cells in either condition.

To examine the functional properties and synaptic integration of cells born after TBI, we performed current-clamp and voltage-clamp recordings from fluorescently labeled adult-born cells in POMC-EGFP mice 2 weeks after CCI. Immature neurons in the sham injury and CCI groups had similar passive membrane properties, including high-input resistance and low capacitance, which is typical of immature cells (Espósito et al., 2005; K^+^-based internal solution: input resistance: sham = 4.3 ± 0.6 GΩ, CCI = 3.6 ± 0.4 GΩ, *p* = 0.59^19^; capacitance: sham = 7.0 ± 0.7 pF, CCI = 9.1 ± 1.2 pF, *p* = 0.42^20^; *n* = 10 cells from two animals each; Cs^+^-based internal solution: input resistance: sham = 17.1 ± 3.1 GΩ, CCI = 25.5 ± 4.5 GΩ, *p* = 0.14^21^; capacitance: sham = 4.9 ± 0.7 pF, CCI = 4.8 ± 0.3 pF, *p* = 0.86^22^; *n* = 7, 8 cells from two mice/group). In current-clamp recordings with a K^+^-based internal solution, neurons born after CCI had similar resting membrane potentials and spiking properties as sham controls, typically with a single low-amplitude action potential during depolarizing current steps, as reported previously for adult-born neurons (Overstreet et al., 2004; Espósito et al., 2005; Fig. 3*A*; 8 of 9 cells with only single spikes during prolonged depolarizing current step after sham, 7 of 10 cells with only single spikes after CCI; resting *V*_m_: sham = −53.6 ± 4.4 mV, CCI = −57.4 ± 3.5mV, *p* = 0.43^23^; spike threshold sham = −30.1 ± 1.6 mV, CCI = −26.6 ± 2.7 mV, *p* = 0.33^24^; spike amplitude: sham = 39.9 ± 4.8 mV, CCI = 53.0 ± 11.0 mV, *p* = 0.24^25^; *n* = 9, 10 cells from two mice/group). The action potentials of immature cells born after CCI were slightly narrower (half-width: sham = 3.1 ± 0.3 ms, CCI = 2.6 ± 0.2 ms, *p* = 0.014^26^) but had similar rise rates (sham = 24.7 ± 3.8 mV/ms, CCI = 40.2 ± 13.3 mV/ms, *p* = 0.34^27^; *n* = 9, 10 cells from two mice/group).

To assess synaptic currents, we made recordings from immature cells with a Cs^+^-based internal solution in voltage clamp at various holding potentials. In these experiments, immature adult-born neurons had similar magnitude fast, voltage-dependent (presumably Na^+^) currents activated by depolarizing voltage steps (Fig. 3*B*,*C*). To stimulate excitatory afferents from entorhinal cortex as well as excitatory associational/mossy cell inputs located in the IML, we positioned a stimulating electrode at the IML/middle molecular layer border. This stimulation also activates direct inhibitory inputs onto granule cells as well as feedforward inhibitory pathways. In all cells assayed (sham, *n* = 8; CCI, *n* = 7), electrical stimulation elicited synaptic currents (Fig. 3*D*) that were almost completely blocked by the GABA_A_R antagonist SR95531 (10 μm; Fig. 3*E*). This result is consistent with prior observations that adult-born granule cells almost exclusively have GABAergic synaptic inputs at this early stage (Espósito et al., 2005; Overstreet Wadiche et al., 2005; Ge et al., 2006). Furthermore, analysis of IPSC decay kinetics demonstrated no difference between groups (sham τ = 138 ± 16 ms; CCI τ = 138 ± 20 ms; *p* = 0.99).

In the presence of GABA_A_R blockade, we elicited small, slowly decaying currents at +40 mV in a proportion of cells in each group (three of seven cells in sham mice, four of seven in CCI mice), which were consistent with prior demonstration of NMDAR-only excitatory synapses in ∼50% of cells in this immature population (Chancey et al., 2013). However, at −70 mV, only one cell (in a post-sham injury mouse, of seven cells assayed in each group) had a small AMPAR-mediated response (10 pA) despite supramaximal stimulation. Although we used a high stimulation intensity to evoke responses, to eliminate the possibility that we failed to excite excitatory afferents due to electrode position, we also looked for sEPSCs. However, none of the cells had more than two putative events in 10 min of continuous recording per cell, with the majority of cells having no events. Thus, we conclude that the sEPSC frequency was extremely low (<0.002 Hz), with no difference in sEPSC frequency between conditions (sham sEPSC frequency = 0.0005 ± 0.0004 Hz; CCI sEPSC frequency = 0.0013 ± 0.0007 Hz; *p* = 0.24^28^; *n* = 7, six cells from two mice/group).

Consistent with the absence of excitatory synaptic activity, there were no dendritic spines detected in GFP^+^ neurons from sham- or CCI-treated mice (Fig. 3*F*,*G*). Thus, although the mild heterogeneity in postmitotic age of GFP^+^ cells (Overstreet et al., 2004) may have obscured minor electrophysiological differences between groups, immature adult-born cells developing post-CCI show the expected early functional maturation of normal adult-born granule cells. This includes the development of intrinsic excitability and GABAergic synapses at an early maturational stage without any precocious maturation of excitatory synapses.

### Persistent survival and ectopic migration of mature neurons born shortly after CCI

Our results to this point with POMC-EGFP-labeled cells indicate that up to 2 weeks post-CCI, cells born after TBI have abnormal dendritic development and migration, but grossly normal electrical and synaptic properties. However, changes that might appear in these cells during their subsequent maturation could not be assessed with this mouse, as cells in POMC-EGFP mice no longer express GFP at 1 month postmitosis. Thus, we used DcxCre/tdTom mice to pulse label the granule neurons born in the first few days following CCI, so that we could analyze this same cohort of cells at a later time point. In these mice, immature adult-born neurons express a tamoxifen-inducible Cre recombinase, CreER^T2^, under the control of the doublecortin promoter (Cheng et al., 2011). These mice were crossed to a Cre-dependent marker mouse (Rosa26-CAG-tdTomato), such that tamoxifen permanently induced tdTomato expression in a cohort of immature cells, allowing them to be examined after they matured. We administered tamoxifen 6-8 d after CCI or sham treatment and assessed labeled neurons at 1 month after CCI.

**Figure 4.**
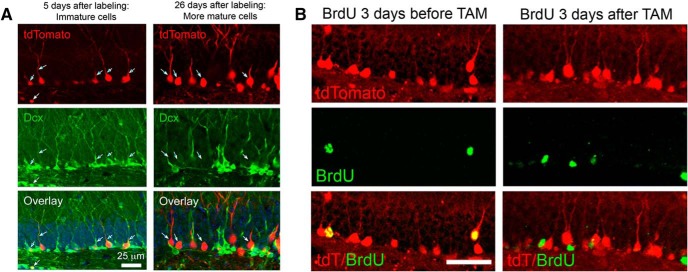
Tamoxifen administration pulse labels adult born neurons in DcxCre/tdTomato mice. ***A***, Tamoxifen (TAM) was administered to adult DcxCre/tdTom mice, and they were fixed at two different time intervals thereafter. Early after TAM administration (left), tdTom^+^ cells have immature dendritic morphologies and stain for the immature marker Dcx, indicating recent mitosis. After 3 weeks (right), tdTom^+^ cells no longer costain for Dcx, suggesting that labeled cells mature, and that no new immature cells have been labeled. The minor overlap of red and green in the projected image at 26 d after TAM administration reflects the overlap of separate cells in the stack and not coexpression. ***B***, DcxCre/tdTom mice received BrdU to mark cells born either before (left) or after (right) adult TAM administration, and were fixed 3 weeks later. Cells born before TAM administration are efficiently labeled by tdTom expression, but cells born after TAM did not express tdTom, indicating that neurons born just prior to tamoxifen administration are selectively pulse labeled. Scale bar, 50 μm.

Although well characterized in the original report (Cheng et al., 2011), we confirmed that tamoxifen administration induced tdTom expression in immature cells by examining Dcx costaining early after tamoxifen treatment (Fig. 4*A*, left), which confirmed that tdTom^+^ cells were all immature adult-born cells at this time point. Over the next 3 weeks, these cells matured *in vivo*, losing Dcx expression and acquiring a more mature morphology (Fig. 4*A*, right). Cells born after tamoxifen treatment were not recruited into the tdTom-labeled pool after tamoxifen withdrawal, based on both the lack of Dcx/tdT costaining at later time points (Fig. 4*A*, right) and the lack of tdTom expression in cells born after tamoxifen as assessed by BrdU colabeling (Fig. 4*B*). Thus, the DcxCre/tdTom mice allowed us to examine the long-term fate of granule cells born during early post-traumatic neurogenesis.

**Figure 5.**
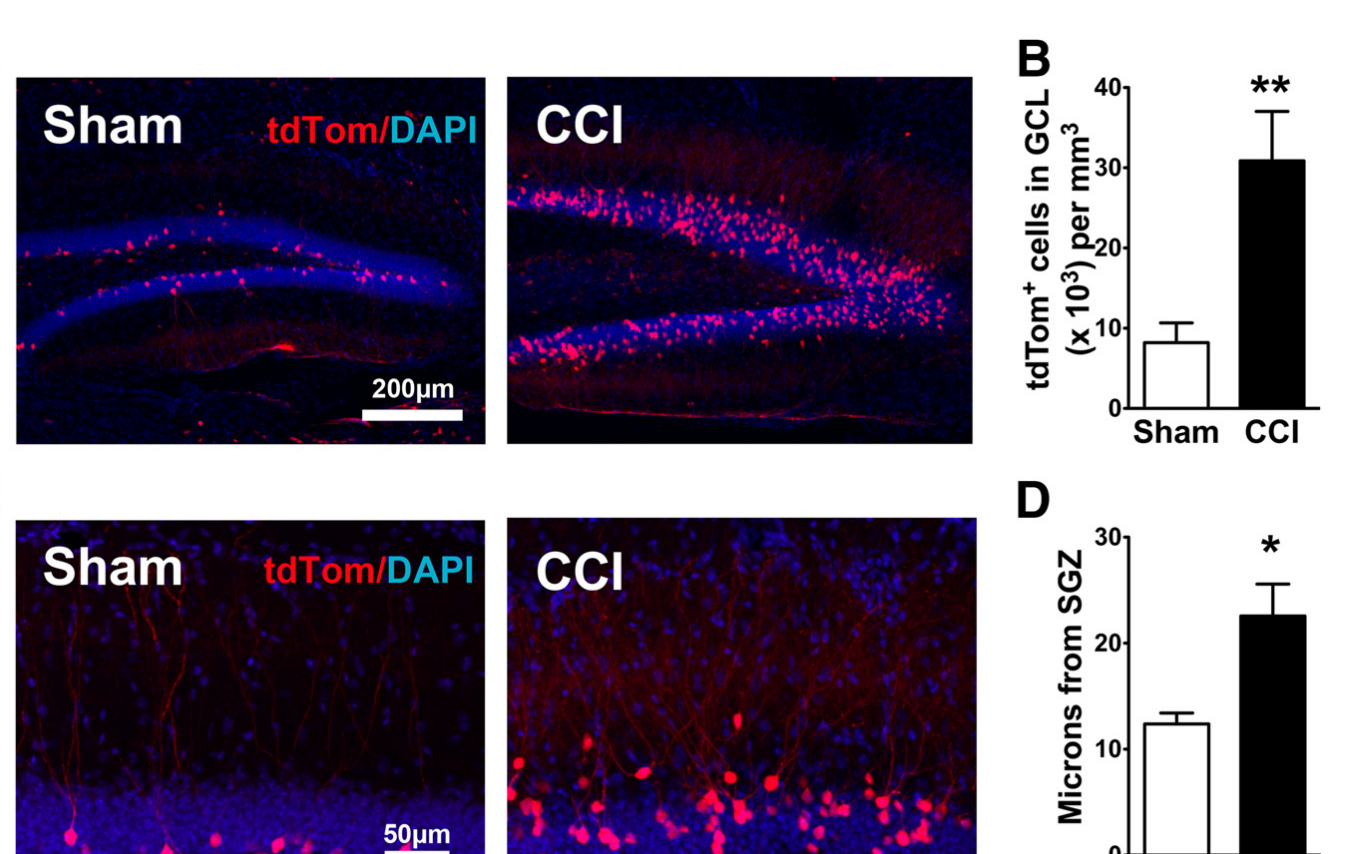
Neurons born shortly after CCI survive and maintain their aberrant localization in maturity. ***A***, Representative images of ∼4-week-old tdTom^+^ cells from the ipsilateral hippocampus of DcxCre/tdTom sham and CCI-treated mice, in which cells born after CCI were permanently pulse labeled with tdTomato. ***B***, One month after injury, CCI-treated mice had more tdTom^+^ cells in the ipsilateral granule cell layer of the dentate gyrus compared with sham mice (**p = 0.0087^29^, *n* = 6 mice/group). ***C***, Representative images of tdTom^+^ cell dispersion in the granule cell layer of the ipsilateral dentate gyrus. ***D***, CCI-treated mice had greater cell migration in the ipsilateral dentate gyrus compared with sham mice (*p = 0.042^30^, *n* = 6 mice/group).

As expected, there were more tdTom^+^ neurons in CCI-treated mice compared to sham mice at this interval (*p* = 0.0087^29^, *n* = 6 mice/group; Fig. 5*A*,*B*). The increase in tdTom^+^ CCI-induced newborn neurons was greater than the increase in CCI-treated POMC-EGFP mice (compare Figs. 1*C*, 5*B*). This difference may have been due to slight differences in the exact cohort of cells sampled in POMC-EGFP mice versus DcxCre/tdTom mice, as there is a biphasic change in neurogenesis following CCI, with an initial reduction followed by a subsequent increase in proliferation in the dentate gyrus (Yu et al., 2008).

The increased outward migration of cells born after CCI (Fig. 1*D–F*) was still apparent at this later time point when compared to matched samples from sham mice (*p* = 0.042^30^, *n* = 6 mice/group; Fig. 5*C*,*D*). The ectopic migration of adult-born neurons into the molecular layer also persisted (*p* = 0.01^31^; tdTom^+^ cells in the molecular layer: sham = 0% ± 0; CCI = 7.1% ± 2.4, *n* = 6 mice/group), suggesting that the ectopically migrated cells survive and maintain their aberrant localization.

### Persistent aberrations in dendritic morphology of mature cells born shortly after CCI

**Figure 6.**
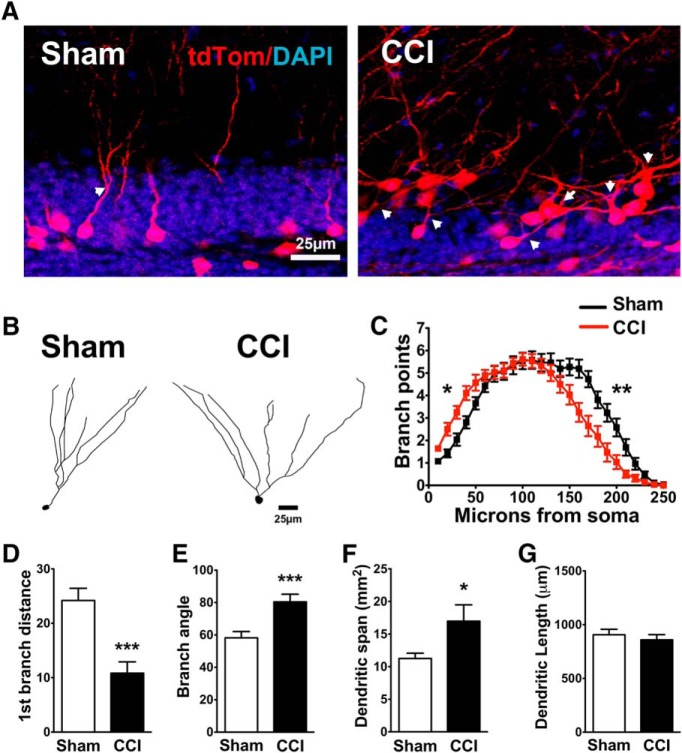
CCI-induced changes in the dendritic morphology of newborn neurons persist as these cells mature. ***A***, Representative images of 4-week-old tdTom^+^ cells from sham and CCI-treated mice. Arrows point to the first dendritic branch point of selected cells. ***B***, Representative tracings of tdTom^+^ cells from sham and CCI-treated mice. ***C***, Sholl analyses of dendritic arborization of tdTom^+^ cells reveal a CCI-induced persistent increase in the number of branch points observed near the soma (*p = 0.0001, 0.003, 0.004, 0.003, and 0.049 for each 10 μm increment between 10 and 50 μm from the soma^34^), but a reduction in more distal regions (**p = 0.038, 0.002, 0.001, 0.009, 0.003, 0.003, 0.004, and 0.038 for each 10 μm increment between 150 and 220 μm from the soma^34^; *n* = 26 cells from 7 sham mice; 28 cells from 8 CCI-treated mice). ***D–F***, tdTom^+^ cells from CCI-treated mice branched closer to their cell body (***p = 0.0001^35^; ***D***), and had wider angles (***p = 0.0007^36^; ***E***) and area (*p = 0.040^37^; ***F***) between the dendrites that were farthest apart. ***G***, tdTom^+^ cells from sham and CCI-treated mice had similar cumulative dendritic lengths (*p* = 0.50^56^).

At 1 month after injury, pulse-labeled tdTom^+^ cells born after CCI continued to display an altered pattern of dendritic arborization, as determined by Sholl analysis (*F*_(29,1560)_ = 5.0; *p* = 0.0001^32^, distance × treatment interaction, two-way repeated-measures ANOVA, *n* = 26 cells from seven sham mice; 28 cells from eight CCI-treated mice; Fig. 6*A–C*). This effect was independent of the location of the cells within the GCL, as a significant effect of CCI was still present when we compared cells from sham and CCI-treated mice that had similar migration distances (*F*_(29,522)_ = 1.615; *p* = 0.021^33^, branch point × treatment interaction, *n* = 9, 11 cells from three mice/group).

Morphologically, tdTom^+^ cells in both the sham and CCI groups had more extensive dendritic trees than GFP^+^ cells in POMC-EGFP mice, which is consistent with their further maturation *in vivo*, as tdTom^+^ cells are ∼1 month postmitosis compared with 7-14 d postmitosis for POMC-EGFP^+^ cells. Similar to immature neurons in POMC-EGFP mice, mature cells born after CCI had more proximal branches (Fig. 6*C*, left side of distance axis on the Sholl plot; *p* = 0.0001, 0.003, 0.004, 0.003, and 0.049 for each 10 μm increment between 10 and 50 μm from the soma^34^), with the first proximal branch occurring closer to the cell soma (*p* = 0.0001^35^; Fig. 6*D*). At farther distances, however, there were fewer branches compared with sham mice (*p* = 0.038, 0.002, 0.001, 0.009, 0.003, 0.003, 0.004, and 0.038 for each 10 μm increment between 150 and 220 μm from the soma^34^; Fig. 6*C*).

**Figure 7.**
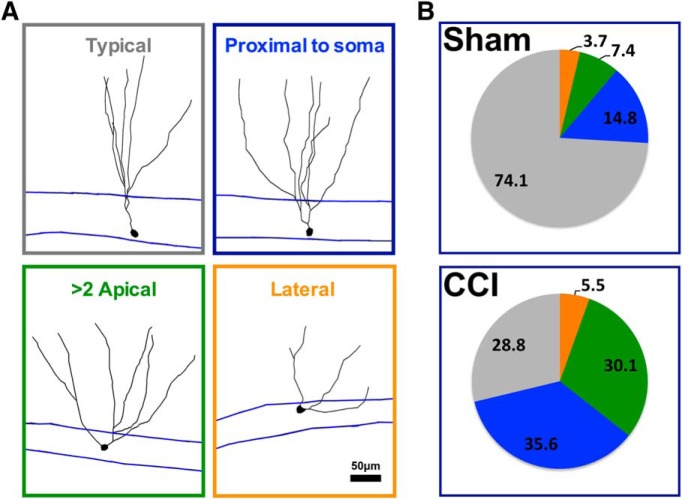
CCI increased the heterogeneity of dendritic branch morphology in cells born after injury. ***A***, Representative morphology of tdTom^+^ cells categorized according to their dendritic phenotype. ***B***, The majority of tdTom^+^ cells in sham mice were characterized by a single apical dendrite that branched farther away from the cell body (>10 μm from soma, typical cell), whereas the majority of tdTom^+^ cells in CCI-treated mice branched more proximal to their somata (<10 μm), had more than one apical dendrite, or had an apical dendrite that projected laterally from their soma (<30° from GCL). There were fewer typical tdTom^+^ cells in CCI-treated mice compared with sham mice (*p* = 0.021^38^, *n* = 5 mice/group).

Interestingly, tdTom^+^ cells from CCI-treated mice had wider angles between primary dendrites (*p* = 0.0007^36^; Fig. 6*E*), and their dendritic arbor covered a wider area of the molecular layer (*p* = 0.0398^37^; Fig. 6*F*; *n* = 26 cells from seven sham mice; *n* = 28 cells from eight CCI-treated mice). Mature granule cells born during post-traumatic neurogenesis also had a notable increase in morphologic heterogeneity (Fig. 7), including cells with lateral projecting dendrites, with more than one apical dendrite, and with a major dendritic branch just adjacent to their soma (<10 μm). This effect was coincident with a decrease in the proportion of cells with “typical” morphology, which was defined as a single apical dendrite directed toward the molecular layer (*p* = 0.021^38^, CCI vs sham mice; *n* = 5 mice per group).

### Neurons born after CCI functionally integrate into the hippocampal circuit 1 month after injury

**Figure 8.**
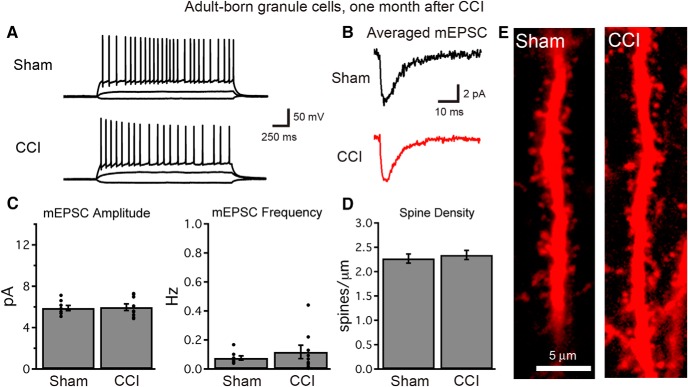
Granule cells born after TBI become functionally integrated into the hippocampal circuit. ***A***, At 1 month after injury, single-cell recordings demonstrate normal firing patterns and the excitability of cells born early after TBI. Voltage traces show the response of adult-born granule cells to current injections of −10, +10, and +50 pA. ***B***, ***C***, Whole cell, voltage-clamp recordings of mEPSCs demonstrate that cells born after TBI acquire excitatory synaptic connections (***B***), and that mEPSC amplitude and frequency (***C***) are similar to those recorded from adult-born cells of similar age in sham animals (*n* = 8 and 9 cells for sham and CCI, respectively; *p* > 0.4 each). Each graph depicts summary data (bars) and individual cell data (points). ***D***, At 1 month after injury, cells born early following TBI possess dendritic spines within the dentate molecular layer, and dendritic spine density is similar in cells born after sham or CCI procedures (sham, *n* = 16 cells; CCI, *n* = 22 cells; *p* = 0.60^53^). ***E***, Images of dendritic spines from cells born after CCI or sham procedure.

Despite the aberrant morphology and outward migration of adult-born cells in mice after CCI, tdTom^+^ cells in acute hippocampal slices in 3-month-old mice (1 month after CCI or sham injury) had the same passive membrane properties as labeled cells in sham animals, as assessed using single-cell recording techniques (K^+^-based internal solution: input resistance: sham = 1080 ± 120 MΩ; CCI = 1450 ± 430 MΩ; *p* = 0.54^39^; capacitance: sham = 37.8 ± 3.0 pF; CCI = 41.2 ± 5.7 pF; *p* = 0.0.68^40^; *n* = 7 from two sham mice, 10 cells from three CCI-treated mice). Likewise, the properties of action potentials evoked by depolarizing current injection (Fig. 8*A*) and resting membrane potentials were similar between groups (resting *V*_m_: sham = −76.0 ± 2.3 mV; CCI = −75.5 ± 2.6 mV; *p* = 0.90^41^; spike threshold sham = −38.7 ± 1.3 mV; CCI = −38.1 ± 1.7mV; *p* = 0.83^42^; spike amplitude from threshold: sham = 121.2 ± 1.3 mV; CCI = 116.2 ± 8.1 mV; *p* = 0.66^43^; *n* = 6, 11 cells from two sham, three CCI-treated mice). By 1 month after injury, cells born after sham injury or CCI did have faster action potentials than this same population of cells when sampled earlier (at 2 weeks after injury in POMC-EGFP mice), but action potential half-width was no longer significantly different (sham = 1.1 ± 0.1 ms, CCI = 1.3 ± 0.3 ms, *p* =0.66^44^; *n* = 6 and 11 cells from two and three CCI-treated mice). Action potential firing rates, spike frequency accommodation, and spike afterhyperpolarization were also similar between cells (data not shown).

To assess the functional synaptic integration of cells born after TBI, we then performed whole-cell, voltage-clamp recordings from tdTom^+^ cells using a cesium-based internal solution to assay synaptic currents at a variety of holding potentials. All cells tested in each group had detectable mEPSCs (Fig. 8*B*). The amplitude and frequency of mEPSCs were similar between conditions (Fig. 8*C*), suggesting that both the strength of individual excitatory synapses as well as the total number of functional excitatory synapses were not different between adult-born cells from each group. The results were similar when sEPSCs were recorded in the absence of TTX, to include action potential-dependent events (average sEPSC amplitude: sham = 6.7 ± 0.5 pA, CCI = 6.9 ± 0.4 pA, *p* = 0.72^45^; sEPSC frequency: sham = 0.10 ± 0.02 Hz, CCI = 0.19 ± 0.06 Hz, *p* = 0.29^46^; *n* = 7 and 11 cells from three mice/group). The sEPSC kinetics also did not demonstrate differences between groups (decay τ: sham = 6.9 ± 0.3 ms; CCI = 6.8 ± 0.5 ms, *p* = 0.18^47^; *n* = 7 and 11 cells). Finally, as the activity of inhibitory circuits can change after TBI (Santhakumar et al., 2001), we examined inhibitory synaptic currents while holding cells at the reversal potential for excitatory currents. Both the frequency and amplitude of sIPSCs was unchanged between cells born after CCI or a sham procedure (sIPSC amplitude: sham = 10.9 ± 0.8 pA, CCI = 10.5 ± 1.8 pA, *p* = 0.88^48^; sIPSC frequency: sham = 0.53 ± 0.28 Hz, CCI = 0.38 ± 0.25 Hz, *p* = 0.70^49^; *n* = 6 and 7 cells from three mice/group). Thus, despite significant changes in the dendritic architecture, cells born during post-traumatic neurogenesis acquire synaptic innervation typical of adult-born neurons at this stage. In accord with these findings, CCI did not alter the dendritic spine density of tdTom^+^ adult-born granule cells (Fig. 8*D*,*E*).

**Figure 9.**
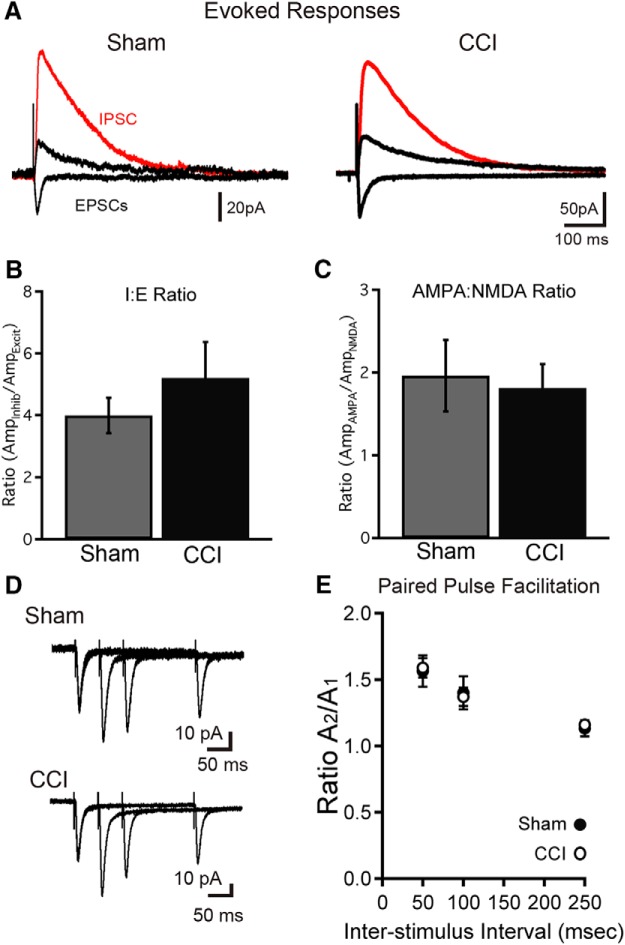
Granule cells born after TBI acquire perforant path inputs and maintain balanced functional connectivity between signaling pathways. ***A***, Stimulation of the dentate molecular layer evokes both excitatory and inhibitory currents in cells born after sham and CCI procedures. Inhibitory currents were recorded while voltage clamping adult-born cells to 0 mV (red traces), and excitatory currents (black) were recorded at −70 and +40 mV in the presence of the GABA_A_R inhibitor SR95531, with the stimulation intensity and electrode position kept constant. ***B***, The ratio of evoked inhibitory current amplitude (at 0 mV) to excitatory current amplitude (AMPAR mediated at −70 mV) was measured for single cells with the same stimulation parameters. This inhibition/excitation ratio (I:E ratio) was similar for cells born after sham or CCI (*p* = 0.42^50^; *n* = 9 and 13 cells). ***C***, The ratio of AMPAR- to NMDAR-mediated currents at perforant path synapses was similar for cells born after CCI (*p* = 0.77^51^; *n* = 11 and 16 cells). ***D***, ***E***, Paired-pulse facilitation was not different at perforant path synapses for cells between groups. ***D***, Example traces show overlaid pairs of responses with interpulse intervals of 50, 100, and 250 ms for cells born after both sham and CCI. ***E***, Summary data show no difference in paired-pulse facilitation between groups (*p* = 0.86^52^, 0.82^52^, and 0.68^52^ for 50, 100, and 250 ms interspike intervals, respectively; sham, *n* = 8; CCI, *n* = 11 cells from 3 mice/condition).

Spontaneous and miniature excitatory synaptic events can arise from synapses located across the entire cell, and sample both mossy cell inputs from the hilus as well as entorhinal afferents. Thus, to focus more specifically on afferent (extrinsic) connectivity, we directly evoked excitatory responses arising from entorhinal afferents as well as inhibitory responses from direct and feedforward inhibitory pathways (Freund and Buzsáki, 1996). Stimulation of the molecular layer evoked excitatory and inhibitory synaptic currents in all cells (Fig. 9*A*). Although neuronal injury can cause an imbalance between excitatory and inhibitory innervation (El-Hassar et al., 2007), cells born after TBI had a normal ratio of excitation to inhibition (*p* = 0.42^50^, *n* = 9 and 13 cells, from three mice/group; Fig. 9*A*,*B*). Cells born after CCI also had a normal ratio of AMPAR/NMDAR-mediated currents (*p* = 0.77^51^; *n* = 11 and 16 cells from three mice/group; Fig. 9*A*,*C*). Finally, paired-pulse facilitation of the evoked EPSC, a measure of presynaptic function, was not different between groups (Fig. 9*D*,*E*). Thus, despite persistent morphologic abnormalities, granule cells born after TBI not only fully integrated into the hippocampal circuit, but maintained intact functional properties.

## Discussion

It is now well established that injuries such as TBI can increase neurogenesis in the dentate gyrus (Dash et al., 2001; Chirumamilla et al., 2002; Villasana et al., 2014). However, the subsequent fate and function of these adult-born granule cells has been challenging to study, as they are anatomically interspersed among an abundance of mature granule cells (Aimone et al., 2014). Retroviral labeling techniques allow for long-term tracing of adult-born cells as well as electrophysiological analysis (van Praag et al., 2002), although this requires a direct intrahippocampal injection of viral particles, which itself induces trauma that could confound analysis. Our use of POMC-EGFP and DcxCre/tdTom transgenic mice allowed us to study the fate of adult-born granule cells early after their generation and weeks later, respectively. Our results indicate that the adult-born granule cells generated post-TBI survive and become integrated into the hippocampal network, thus leading to a net increase in the cohort of functional new neurons born following TBI. Neurons born after TBI acquired typical levels of synaptic innervation, and maintained a balance between excitatory and inhibitory synaptic activity that was similar to that of age-matched adult-born neurons from sham mice.

In addition to the increased production of new neurons after injury, we observed that CCI altered the migration and morphologic development of cells born after injury. Changes in the migration and dendritic development of adult-born neurons have also been observed following other types of brain injuries, including experimental stroke (Niv et al., 2012), neonatal hypoxia (Pugh et al., 2011), entorhinal cortical lesions (Perederiy et al., 2013), and seizures (Scharfman et al., 2000; Jessberger et al., 2007; Shapiro et al., 2008). However, changes in the development of adult-born neurons appear to be injury specific. For example, experimental temporal epilepsy and stroke respectively increase the development of spines and spine density of newborn neurons born after injury (Overstreet-Wadiche et al., 2006; Niv et al., 2012). In contrast, we did not find accelerated development of spines in young neurons born after CCI or changes in the spine density of older CCI-induced neurons. Additionally, unlike experimental stroke and seizure (Parent et al., 1997; Shapiro et al., 2008; Niv et al., 2012), we did not observe an increase in the formation of basal dendrites by adult-born neurons generated after CCI.

The effect of TBI on the generation and development of adult-born neurons might also be modulated by the degree of injury. For example, a recent study by Carlson et al. (2014) reported that dendrites of immature cells born after TBI were stunted, as determined by their Dcx staining pattern. However, their model often caused direct damage to the hippocampus, and, in contrast to most other studies, decreased neurogenesis after injury. In our own work, slices in which the hippocampus was directly damaged displayed a massive increase in necrotic cell death within the dentate, which was not as obvious in our immunohistochemical experiments but was very clear in acutely prepared slices (data not shown). As we were more interested in the development of newborn granule cells in an otherwise intact hippocampus, we specifically excluded these slices, and implemented a model in which this rarely occurred. Thus, there are distinctions in the manner in which different types and severities of brain injuries affect the development of adult-born neurons.

### Implications for post-traumatic neurogenesis

Due to its therapeutic implications, there has been growing research interest in promoting post-traumatic neurogenesis as an adjunctive treatment to restore cognitive function lost as a consequence of injury (Lu et al., 2003; Lu et al., 2005; Quadrato et al., 2014; Chohan et al., 2015; Xie et al., 2015). Post-traumatic neurogenesis is mainly thought to be beneficial, based on the positive association between neurogenesis and several types of learning (Shors et al., 2001; Clelland et al., 2009; Sahay et al., 2011; Jackson et al., 2012; Nakashiba et al., 2012) and the observation that manipulations that alter neurogenesis after injury affect outcomes in animal models (Lu et al., 2005; Sun et al., 2009; Blaiss et al., 2011; Sun et al., 2015). However, as positive modulators have other effects, and the inhibition of neurogenesis is itself detrimental to learning (Shors et al., 2001; Clelland et al., 2009; Nakashiba et al., 2012), these results are difficult to interpret. If cells born after injury have aberrant functional integration, they might actually contribute to ongoing brain dysfunction, as has been suggested previously in other injury contexts (Parent and Lowenstein, 2002; Scharfman and Hen, 2007). A future analysis of the network-level effects of the neurons generated after injury will hopefully help to answer this question.

Although we did not detect physiological differences at the cell-intrinsic or overall synaptic level, the altered dendritic fields and increased proximal weighting of dendritic branch density observed in the cells born after TBI could increase the relative weighting of inputs onto these cells from the inner molecular layer (primarily mossy cell and other intrinsic hippocampal inputs) relative to those in the outer molecular layer (extrinsic lateral entorhinal afferents). As these particular input streams carry different information (Lisman, 2007), the reweighting of these inputs could have profound functional implications at the network level. Although our work does not specifically address whether the changes noted in cells born during post-traumatic neurogenesis are beneficial or maladaptive, it does demonstrate that the neurons generated after injury have both similarities and differences from those generated in healthy brains, and future work will help to further define their functional parameters and contribution to recovery, ongoing pathology, or both.*-1687, USA 2Harvard*

## Synthesis

The decision was a result of the Reviewing Editor Pablo Castillo and the peer reviewers coming together and discussing their recommendations until a consensus was reached. A fact-based synthesis statement explaining their decision and outlining what is needed to prepare a revision is listed below. The following reviewers agreed to reveal their identity: Amar Sahay, Shaoyu Ge

Your manuscript has been evaluated by two reviewers who agree on the good quality of the results and overall merit of your study. Below you will find a synthesis of their comments and online discussion.

Fig 1A, the authors should put together a new set of image showing successful stitching.

Fig 1A-D, the CCI images suggest that non-matching sections of the dentate gyrus were used, raising concerns on the analysis presented in panels E,F. Similar concerns apply to the images presented in Figs 4 and 5.

Fig 1C legend: CCI treated mice had more GFP+cells on the injured hemisphere compared to sham mice (**P = 0.002, n = 6 mice/group).

Text: CCI increased the number of immature adult-born neurons (GFP+ cells) (F1,26 = 12.09; P = 0.002, n = 7, 8 mice per group; Figure 1B,C).

Fig 3, potential functional differences may have been lost by blending neurons of diverse age.

Fig 4, analyze tdTom+ cells in ML as well.

Figs 5,6, neurons look very different from those presented above (e.g. Fig. 2), suggesting that authors did not pay close attention to the precise anatomical localization of the new born neurons.

Fig 7, the analyzed neurons are already mature and therefore, the analysis is not very informative; the authors should analyze younger neurons as well.

Show the data confirming the utility of the regimen used to induce tdTomatoe expression in new neurons in the DCX CreERT2 mice.

eN-NWR-0056-15R1 Response to reviewers

We were very pleased that the reviewers felt that our experiments were sound and clean, and that our results were interesting. The reviewers' comments primarily concerned data presentation, and in each instance we have either revised our presentation of the data, or added additional clarification to the text and/or figure legends to allow for easier interpretation of our data. We have also added additional data in both Figure 1 and a new Figure 4, as well as in the text. We address each on a point-by-point basis below.

Synthesis of Reviews:

Your manuscript has been evaluated by two reviewers who agree on the good quality of the results and overall merit of your study. Below you will find a synthesis of their comments and online discussion.

This manuscript reports that traumatic brain injury (TBI) alters the anatomical localization and dendritic complexity, but not basic functional properties of adult-born dentate granule neurons. These findings are interesting and represent a first thorough characterization of TBI-induced effects on these neurons. Overall, the experiments are well designed, the results are clean and the conclusions are sound. However, the reviewers raised some concerns regarding the data presentation and analysis that should be properly addressed.

Fig 1A, the authors should put together a new set of image showing successful stitching.

- We have re-captured these images to improve the stitching and have amended Figure 1A accordingly.

Fig 1A-D, the CCI images suggest that non-matching sections of the dentate gyrus were used, raising concerns on the analysis presented in panels E,F. Similar concerns apply to the images presented in Figs 4 and 5.

- For both the figures and the analysis, sections of dentate gyrus were matched between sham and CCI animals, using slices obtained from the same position on the rostral/caudal axis for coronal sections as stated in the methods. Similarly, all ipsi/contralateral analyses were performed on the same coronal sections. However, sections immediately adjacent (ipsilateral) to the injury sometimes demonstrated morphologic distortion of the dentate, as is seen in the ipsi/contralateral sections in Figure 1A. We did consider that this morphologic distortion might itself have an effect on neurogenesis and cell migration, so we repeated our analyses on the more ventral regions of hippocampus far from the injury, in which no gross morphologic changes to the dentate were apparent. The CCI effects persisted, indicating that the effects of CCI on neurogenesis are not secondary to anatomic distortion of the hippocampus. This observation is referenced in the text (page 11). We have amended the results text to emphasize that these sections were taken from matching regions of the dentate, and have added images from ventral hippocampus (now Figure 1C) to illustrate the quantification that was already in the text.

Fig 1C legend: CCI treated mice had more GFP+cells on the injured hemisphere compared to sham mice (**P = 0.002, n = 6 mice/group). Text: CCI increased the number of immature adult-born neurons (GFP+ cells) (F1,26 = 12.09; P = 0.002, n = 7, 8 mice per group; Figure 1B,C).

- The statistics mentioned in the figure legend were incorrect and have been corrected. In the text, we have also clarified the particular statistical tests used to compare sham and CCI animals, as we performed two separate tests in order to place our results in context with prior results. Specifically, although an ANOVA suggests that there is no injury by hemisphere interaction, both in our work and throughout the literature, the effect of CCI on neurogenesis is more marked in the ipsilateral hemisphere, and often exclusively analyzed on that side. As it was our a priori intention to focus our morphologic and physiologic analyses on the ipsilateral side, we examined each side in isolation using t-tests, in addition to using an ANOVA to analyze the interaction of hemisphere and injury.

Fig 3, potential functional differences may have been lost by blending

neurons of diverse age.

- The GFP+ adult-born cells labeled in POMC-eGFP mice are 7-14 days postmitosis, thus somewhat heterogeneous in their exact ages. Cells continue to mature during this timeframe, thus it is correct that GFP+ cells at 14 days post-mitosis might have different functional parameters than the younger GFP+ cells. However, in both sham and CCI conditions, we were equally likely to sample a mixture of younger and older cells from within the GFP+ pool, and we believe that a systematic acceleration of maturation of any of the parameters studied would still have been detectable. However, given the variability among the cells within each group, we may have missed a subtle difference between groups. We have amended our results to draw attention to the variability inherent in this technique as well as the possibility that subtle differences may have gone undetected. At the same time, we can say with confidence that unlike epileptic seizures, which cause a precocious development of dendritic spines and functionally detectable excitatory synapses onto POMC-eGFP cells in the same mouse strain, we do not see accelerated excitatory innervation in post-CCI animals. A more temporally precise method for dating adult-born cells, such as retroviral injection/labeling, would be inappropriate to pursue given the brain trauma from the injection itself.

Fig 4, analyze tdTom+ cells in ML as well.

- We have performed this analysis and the results have been placed in the text (Page 16).

Figs 5,6, neurons look very different from those presented above (e.g. Fig. 2), suggesting that authors did not pay close attention to the precise anatomical localization of the new born neurons.

- The cells in Figures 5,6 are taken from the same anatomical location (dorsal hippocampus) as those in Figure 2. Their morphology is different primarily because the cells in Figures 5,6 are approximately 2-3 weeks more mature than the cells in Figure 2. As expected, cells at this later stage have more extensive and longer dendritic trees. We specifically used these two different mouse lines to analyze the cells born after CCI at two different timepoints in their maturation, so that we could detect any injury-induced changes in their early maturation as well as determine whether there are any long-lasting changes in their properties. We have clarified the different timepoints used in the text as well as the figure legend.

Fig 7, the analyzed neurons are already mature and therefore, the analysis is not very informative;

- We respectfully disagree that this analysis is not informative, as this is the first functional demonstration that neurons born during post-traumatic neurogenesis become synaptically integrated in the brain and that they retain many functional properties of constitutively generated adult-born neurons. This observation is critical to hypotheses of the involvement of adult-born neurons in recovery after injury.

the authors should analyze younger neurons as well.

- Our physiologic analyses of this population of neurons at an earlier (younger) timepoint is shown in Figure 3 and described in the text on Pages 13-15. We performed this earlier analysis using POMC-eGFP mice, as the tamoxifeninduced tdTomato expression is only weakly visible at the end of the tamoxifen pulse, so it is not ideal for visualizing cells or recording from them at earlier stages in their maturation. This point was clarified in the text.

Show the data confirming the utility of the regimen used to induce tdTomatoe expression in new neurons in the DCX CreERT2 mice.

- We used the same dosage of tamoxifen as the original authors reported with the creation of these mice (Chen et al., 2010; citation in text), although we used only 3 days of tamoxifen rather than 5 days in order to narrow the labeling time window. We have confirmed that our regimen specifically pulse labels adult-born cells using Dcx co-staining as well as BrdU administration/staining. Early after the tamoxifen pulse, almost all tdTom+ neurons are Dcx-positive, suggesting that they were born in the preceding weeks. This Dcx co-staining is completely gone in tdTom+ cells after three weeks, consistent with the cells' maturation. Similarly, BrdU labeling of mitotic cells demonstrated that cells born just before tamoxifen administration are well labeled by tdTomato, but that cells born after tamoxifen withdrawal are not. Together this data confirms that our regimen selectively pulse-labels adult-born granule cells similar to the original report on these animals. We have placed this data into the text and added an additional figure (Figure 4) to display these results.
